# Expert consensus‑based clinical practice guidelines for nutritional support in the intensive care unit: the French Intensive Care Society (SRLF) and the French-Speaking Group of Pediatric Emergency Physicians and Intensivists (GFRUP)

**DOI:** 10.1186/s13613-025-01509-0

**Published:** 2025-07-15

**Authors:** Jean Reignier, Benedicte Gaillard-Le Roux, Pierre François Dequin, Valeria A. Bertoni Maluf, Julien Bohe, Michael P. Casaer, Agathe Delbove, Claire Dupuis, Eric Fontaine, Prescillia Gamon, Coralie Grange, Nicholas Heming, Melissa Jezequel, Adam Jirka, Corinne Jotterand Chaparro, Michael Landais, Nolwenn Letouze, Claire Morice, Olivier Pantet, Julie Pellecer, Gael Piton, Shancy Rooze, Julie Starck, Jean-Marc Tadie, Fabienne Tamion, Ronan Thibault, Frédéric Valla, Thierry Vanderlinden, Arnaud W. Thille, Nadia Aissaoui

**Affiliations:** 1 Movement-Interactions-Performance (MIP), UR 4334; Nantes University, Nantes, France; 2 Pediatric Intensive Care Unit, Nantes University Hospital, Nantes, France; 3 Intensive Care Unit, Inserm U1100, Bretonneau University Hospital, Tours, France; 4 Department of Nutrition and Dietetics, Geneva School of Health Sciences, HES-SO University of Applied Sciences and Arts Western Switzerland, Carouge, Switzerland; 5 Medical Intensive Care Unit, Lyon Sud University Hospital, Claude Bernard Lyon 1 University, Hospices Civils de Lyon, Lyon, France; 6 Clinical Department and Laboratory of Intensive Care Medicine, Department of Cellular and Molecular Medicine, University Hospitals Leuven, Catholic University Leuven, Leuven, Belgium; 7 Medical-Surgical Intensive Care Unit, CHBA Vannes, Vannes, France; 8 Medical Intensive Care Unit, Gabriel Montpied University Hospital, Clermont-Ferrand, France; 9 Human Nutrition Unit, Clermont Auvergne University, INRAe, CRNH Auvergne, Clermont-Ferrand, France; 10 Basic and Applied Bioenergetics Laboratory, Joseph Fourier University, Grenoble, France; 11 Medical Intensive Care Unit, Grenoble University Hospital, La Tronche, France; 12 Department of Anesthesiology and Critical Care and Burn Unit, Saint-Louis University Hospital, AP-HP, Paris, France; 13 Department of Intensive Care, Raymond Poincaré University Hospital, AP-HP, IHU Prometheus, Versailles Saint Quentin University-Paris Saclay University, Garches, France; 14 Cardiac Intensive Care Unit, Saint-Brieuc Hospital, Saint-Brieuc, France; 15 Hepato-Gastroenterology and Digestive Oncology, IMAD, Nantes University Hospital, ​Nantes, France; 16 Medical-Surgical Intensive Care Unit, Community Hospital Centre, Le Mans, France; 17 Pediatric Intensive Care Unit, Caen University Hospital, Caen, France; 18 Pediatric and Neonatal Intensive Care Unit; Pediatric Specialties Division, Department of Pediatrics, Gynecology and Obstetrics, University of Geneva, Geneva, Switzerland; 19 Department of Intensive Care Medicine, Lausanne University Hospital, Lausanne, Switzerland; 20 Université Paris-Saclay, Faculté de Médecine, Service de Medecine Intensive-Reanimation, Hôpital de Bicêtre, AP-HP, DMU 4 CORREVE Maladies du cœur et des Vaisseaux, Le Kremlin-Bicêtre, France; 21 Medical Intensive Care Unit, SINERGIES, Besançon University Hospital, Franche Comté University, Besançon, France; 22 Paediatric Intensive Care Unit, Hôpital Universitaire de Bruxelles (H.U.B), Université Libre de Bruxelles, Bruxelles, Belgium; 23 Neonatal and Pediatric Intensive Care Unit, Trousseau University Hospital, AP-HP, Paris, France; 24 Intensive Care Unit, Pontchaillou University Hospital, UMR 1236, Rennes University, INSERM, Etablissement Français du Sang Bretagne, Rennes, France; 25 Medical Intensive Care Unit, Rouen University Hospital, Rouen, France; 26 INSERM U1096, Rouen University, Rouen, France; 27 Department of Endocrinology-Diabetology-Nutrition, Home Parenteral Nutrition Centre, CHU Rennes, Rennes, France; 28 INRAE, INSERM, Univ Rennes, Nutrition Metabolism Cancer NuMeCan, Rennes, France; 29 Pediatric Intensive Care, Women’s and Children’s Hospital, Hospices Civils de Lyon, Lyon-Bron, France; 30 Intensive Care Unit, Lille Catholic Institute Hospital Group, FMMS - ETHICS EA 7446, Lille Catholic University, Lille, France; 31 Intensive Care Unit, Poitiers University Hospital, Réanimation, Poitiers, France; 32 Cardiology Department, European Georges Pompidou University Hospital, AP-HP, Paris Cité University, Paris, France; 33 Service de Médecine Intensive Réanimation, Centre Hospitalier Universitaire Hôtel-Dieu, 30 Bd. Jean Monnet, 44093 Nantes Cedex 1, France

**Keywords:** Critical illness, Adults, Children, Nutritional support, Enteral nutrition, Parenteral nutrition, Calories, Proteins, Recommendations

## Abstract

The objective of this work was to develop guidelines for nutritional support in critically ill adults and children (excluding neonates and burn patients) unable to maintain an adequate oral intake. We aimed to provide up-to-date recommendations based on high-level evidence including the results of recent landmark randomized controlled trials. Experts from the French Intensive Care Society (SRLF), the French Society of Clinical Nutrition and Metabolism (SFNCM), and the French-Speaking Group of Pediatric Emergency Physicians and Intensivists (GFRUP) used the GRADE methodology to develop the guidelines. Twenty-four Patient Intervention Comparator Outcome (PICO) questions were identified, resulting in 34 adult and 29 pediatric recommendations. Of the 34 recommendations for adults, three were based on high-level evidence, 12 on moderate-level evidence, and 19 on expert opinion. The corresponding numbers for the 29 pediatric recommendations were one, five, and 23. All recommendations achieved strong agreement among the experts. These guidelines emphasize the importance of individualized nutritional support strategies that incorporate recent high-quality evidence to optimize the outcomes of critically ill patients.

## Introduction

Critical illness is characterized by metabolic and physiological abnormalities including excessive catabolism with protein loss, leading to immune-function impairments and muscle wasting [1–3]. Muscle loss is exacerbated by prolonged immobility, which results from both the neurological effects of critical illness and the sedation required for patient care and comfort. [4]. Optimal nutrition is essential to counteract the effects of excessive catabolism. Inadequate nutritional support has been associated with impaired wound healing, immune dysfunction, secondary infection, aggravated metabolic disturbances, increased muscle loss, higher mortality, and impaired recovery in survivors [5]. Energy, nutrients, and micronutrients must therefore be provided.

Several issues regarding nutritional support for critically ill patients remain debated, notably the optimal amounts of energy and protein to be provided; the best time schedule for the initiation and progression of nutrient delivery; and the choice between, or combination of, parenteral and enteral feeding. Given these complex metabolic challenges, evidence-based guidelines are essential and must evolve in response to emerging evidence. The French Society of Critical Care issued guidelines for the management of nutrition in 2014. However, at the time, the available data consisted chiefly of observational studies providing low-level evidence. Since then, randomized controlled trials (RCTs) have produced new information, notably regarding the acute phase of critical illness, and have also challenged several previous beliefs [6]. Updated guidelines are therefore needed.

The purpose of this work was to develop guidelines informed by recent data, including high-level evidence from RCTs, for the nutritional support of critically ill children and adults. These guidelines are intended for healthcare professionals involved in providing nutrition care to critically ill adults and children (intensivists, dietitians, pharmacists, internists and family physicians). They are also intended for researchers studying nutrition and critical illness and for hospital committees evaluating nutritional support policies. Finally, these guidelines may prove useful as an educational resource for students, healthcare professionals, and the public, including patients and their families.

## Methods

In these guidelines, nutritional support is defined as the provision of enteral nutrition (EN) or parenteral nutrition (PN) to adult (≥ 18 years) and pediatric patients admitted to intensive care units with severe critical illness precluding adequate oral feeding. Neonates and burn patients were excluded. The guidelines were developed by twenty-four experts in adult and pediatric nutrition belonging to the French Intensive Care Society (*Société de Réanimation de Langue Française*, SRLF), the French Society of Clinical Nutrition and Metabolism (Société Francophone de Nutrition Clinique et Métabolisme, SFNCM) and French-Speaking Group of Pediatric Emergency Physicians and Intensivists (*Groupe Francophone de Réanimation et d’Urgences Pédiatriques*, GFRUP), respectively. The experts used the Grading of Recommendation Assessment, Development and Evaluation (GRADE) methodology.

The steering committee identified key questions for both adult and pediatric populations, using the Patient Intervention Comparator Outcome (PICO) format for each. These questions were validated by the experts task force and determined the scope of the literature search. For this search, the task force of experts defined key indexing terms, the time limits, the target population, and the specific outcomes. According to the GRADE methodology, a level of evidence was first assigned to each study identified by the search, based on study design and methodological quality. The experts then determined the overall level of evidence for each PICO question according to the level of evidence of each available study, the consistency of the results between the studies, and the risk/benefit ratio. A high overall level of evidence led to a “strong” GRADE 1 recommendation (i.e., “should be done” or “should not be done”) and a moderate or a low level of evidence to an “optional” GRADE 2 recommendation (i.e., “should probably be done” or “should probably not be done”). In the absence of evidence, the issue was recommended in the form of an expert opinion.

Recommendations developed by the experts were discussed during two meetings of the full panel of experts. Then, experts rated individually each recommendation on a scale of 1 (complete disagreement) to 9 (complete agreement). 1 to 3 reflected a disagreement with the recommendation, 4 to 6 indecision, and 7 to 9, an agreement with the recommendation. All experts voted on both the adult and the pediatric recommendations. A recommendation was considered approved if at least 50% of the experts agreed and no more than 20% disagreed. Strong agreement was defined as agreement by at least 70% of the experts. When strong agreement was not achieved, the recommendation was revised and subjected again to the rating process. Only expert opinions that obtained a strong agreement were finally adopted.

## Results and guidelines

The experts identified 24 PICO questions leading to 34 and 29 recommendations for adults and children, respectively. For adults, the level of evidence was high (GRADE 1) for three recommendations, moderate (GRADE 2) for 12 recommendations, and very low (expert opinion), for 19 recommendations. The corresponding numbers for children were one, five, and 23. Strong agreement by the panel of experts was achieved for all recommendations.

### Guidelines for adults

***Initiation of nutritional support***

**R1:** The experts suggest early initiation of nutritional support in critically ill adults, within 48 h after intensive care unit (ICU) admission.

***Expert opinion, strong agreement***

Critical illness induces structural and functional alterations of the gastrointestinal tract that contribute to cause organ failures [7, 8]. Early enteral nutrition may help mitigate these alterations [8–10]. Observational studies reported that initiating early EN within 24–48 h after ICU admission was associated with lower mortality, shorter invasive mechanical ventilation (MV) duration, and shorter ICU and hospital lengths of stay (LOS) [11–13]. However, data on its impact on ventilator-associated pneumonia (VAP) risk remain conflicting [11, 12]. In patients receiving vasopressor support, early EN was not associated with improved outcomes compared to delayed EN [14, 15]. Meta-analyses comparing early and delayed EN have yielded mixed results. One meta-analysis reported significantly fewer infections, but no difference in mortality with early EN vs. delayed EN [16], while another found that early EN initiated within 24 h after ICU admission was associated with reduced mortality and incidence of pneumonia [17]. There is no clear evidence that early EN is better than early parenteral nutrition. In a study using a marginal structural Cox model and a large prospective database of patients with shock receiving invasive MV, both early EN and early PN were associated with lower day-28 mortality (hazard ratio [HR], 0.89; 95%CI − 0.81 to 0.98, *P* = 0.01) [18]. Last, two recent RCTs comparing early EN to early PN found no differences in mortality or infections [14, 15]. Overall, these data support beneficial effects of early nutrition given enterally or parenterally.

**R2:** In critically ill adults, either enteral nutrition (EN) or parenteral nutrition (PN) can be used within the first week after ICU admission.

***Grade 1+, strong agreement***

This recommendation is based on two large multicenter RCTs comparing early EN to early PN, started within 48 h after ICU admission, continued for one week, and delivering a mean of 20 kcal/kg/d [19, 20]. The CALORIES trial (n = 2388; 83% on MV and 82% on vasoactive drugs) showed no significant difference in day-30 or day-90 mortality, ICU-acquired infections, or ICU LOS; vomiting was more common with EN [19]. A medico-economic sub-study of CALORIES showed that early PN resulted in a negative incremental net benefit at one year [21]. The NUTRIREA-2 trial included 2410 patients receiving MV and vasoactive drugs for shock. The early EN and early PN groups were not different for MV duration, ICU LOS, nosocomial infections, day-28 mortality, or day-90 mortality [20]. Early EN was associated with higher frequencies of vomiting, diarrhea, and bowel ischemia compared to early PN. This recommendation differs from previous guidelines on this point. Most of the earlier guidelines were issued before publication of the CALORIES and NUTRIREA-2 trials and relied on meta-analyses suggesting fewer infections (but similar mortality) with EN. These meta-analyses included studies that produced very low-level evidence, enrolled heterogenous populations, used various definitions of early EN, were not focused on comparing early EN and early PN, varied regarding the total energy supply, and/or had an observational design [16, 22–27].

***Nutritional needs***

**R3:** Given the lack of data on patient outcomes, the experts cannot recommend using a specific equation instead of the standard method for estimating energy needs in critically ill adults (Kcal/kg/d adjusted for body-mass index).

***Expert opinion, strong agreement***

The many equations available for estimating energy expenditure have only 40%–75% accuracy vs. indirect calorimetry (IC) [28–30]. Among them, none has consistently performed better than the others in critically ill patients. A major limitation is their reliance on static variables (age, height, weight, and sex), which do not reflect the changes associated with critical illness [31]. More specifically, metabolic shifts occur during the various phases of critical illness. Thus, weight, the number and severity of organ failures, medications and other treatments, and body temperature change over time. These factors significantly influence energy expenditure yet are not accounted for in the available equations [32]. Furthermore, the accuracy of equations is particularly low in obese and underweight patients [33, 34]. Another major concern is that these equations were validated primarily by physiological studies in specific populations whose features differed substantially from those of critically ill patients [29]. Due to their simplicity, weight-based equations are widely used. However, in patients with fluid overload (e.g., post-resuscitation, edema), the dry or usual body weight should be used in the equations, to improve accuracy. Methods for correcting weight-based calculations by adjusting for the body-mass index (BMI) differ regarding the thresholds and correction factors. However, no studies have compared the effects on patient outcomes of equation-based vs. weight-based energy-intake strategies in critically ill patients. Consequently, given the lack of outcome-based evidence, the experts cannot recommend the use of a specific equation instead of the standard weight-based formula (Kcal/kg/d, adjusted for BMI).

**R4:** Although reliable for assessing energy expenditure, indirect calorimetry (IC) should probably not be used routinely at the bedside with the goal of improving outcomes of critically ill adults.

***Grade 2-, strong agreement***

Predictive equations derived from healthy individuals are inaccurate for estimating resting energy expenditure (REE) in critically ill patients [35–37]. IC, although more reliable, is often difficult to perform in the ICU due to factors such as a high fraction of inspired oxygen, air leaks, and inter-machine variability. Moreover, the measurements must be repeated frequently given the rapidly changing physiology of critically ill patients. Importantly, IC does not account for endogenous nutrient release, which is unaffected by exogenous nutrition [38, 39].

Studies of IC-guided nutritional therapy have yielded mixed results. Meta-analyses suggested either lower mortality or no significant benefit [40–42]. In the Supplemental PN study, adding PN to supply 100% of the energy target calculated by IC from days 4 to 8 was associated with fewer nosocomial infections compared to supplying less than 60% of the energy target by EN alone [43]. The TICACOS and EAT-ICU trials found that IC-guided nutrition failed to significantly improve mortality or long-term physical function, respectively [44, 45]. The TICACOS international study was terminated prematurely due to slow recruitment, highlighting the practical difficulties of implementing IC in daily ICU practice [46]. In summary, despite having theoretical advantages, IC-guided nutrition has not consistently demonstrated clinical benefits [37]. One possible explanation is that measured REE may not reflect energy needs during the acute phase of critical illness. Recent evidence that low-energy feeding is beneficial has decreased the relevance of accurate REE measurement during the acute phase. Whether IC-guided nutrition might improve outcomes of patients with long ICU stays deserves further research. Continued research is also needed to determine whether advances in IC technology improve both accuracy and feasibility in the ICU.

**R5.1:** A low energy supply of 6–8 kcal/kg/d (adjusted for BMI) should probably be administered during the first ICU week in mechanically ventilated patients, instead of the standard energy supply of 20–25 kcal/kg/d (adjusted for BMI).

***Grade 2+, strong agreement***

**R5.2:** The experts suggest changing to a standard energy supply (20–30 kcal/kg/d, adjusted for BMI) at the end of the first ICU week.

***Expert opinion, strong agreement***

Eleven RCTs, including four multicenter trials, compared low-energy to standard-energy nutrition (20–25 kcal/kg/d) [45, 47–56]. In four of these trials, the energy supply was less than 10 kcal/kg/d in the low-energy groups [48–50, 56]. The study period was the entire ICU stay in two trials [45, 56]. Of note, the standard supply in three trials was less than 20 kcal/kg/d, resulting in only a small difference with the low-energy group [47, 50, 52]. For two trials, the control group received 13–17 kcal/kg/d; the supply of 20–25 kcal/kg/d in the other group was achieved using either guidance by repeated IC or intensive nutrition [45, 52]. All trials but two (EDEN and NUTRIREA-3) hypothesized that low-energy nutrition might adversely affect patients. The primary outcome was ICU LOS in two trials (EPaNIC and NUTRIREA-3), quality of life in the EAT trial, and mortality in the other trials. NUTRIREA-3, EPaNIC, and EDEN involved low protein supplies during the first ICU week [48–50]. EPaNIC and NUTRIREA-3 showed significantly shorter ICU stays in the group given low-energy and low protein nutrition [48, 49]. A single-center study (INTACT) was terminated prematurely due to higher hospital mortality in the group given the highest energy intake [52]. In trials that used EN, diarrhea/constipation, vomiting, and mesenteric ischemia (secondary outcomes) were less common with low-energy nutrition, although the differences were not statistically significant [47, 49, 50, 54]. Extending low-energy nutrition beyond the first week produced the same outcomes as did switching to standard nutrition. Trials with follow-ups of at least 6 months found no differences between the low-energy and standard-energy groups [3, 57, 58].

**R6:** In critically ill adults who are receiving standard energy supplies and have hypophosphatemia (< 0.65 mmol/L), the energy intake should probably be reduced to 20 kcal/h (480 kcal/day) for two days, then increased progressively.

***Grade 2+, strong agreement***

Refeeding syndrome is a potentially life-threatening metabolic disorder that occurs when malnourished patients return to normal or near-normal macronutrient intakes. The manifestations consist of electrolyte imbalances with hypophosphatemia, hypokalemia, and hypomagnesemia; hyperglycemia with insulin resistance; and water retention [59]. Hypophosphatemia, considered a hallmark manifestation, results from increased intracellular phosphorylation due to marked cellular anabolism. Differentiating refeeding syndrome-related hypophosphatemia from tubulopathy-induced hypophosphatemia is crucial, as only the former leads to acute symptoms. The management involves phosphorus supplementation and energy restriction to limit cellular anabolism. A single-blinded multicenter RCT included 327 critically ill patients whose serum phosphate levels were < 0.65 mmol/L within 48 h after starting nutritional support [60]. Patients were randomized to standard care or to the intervention consisting in decreasing the energy intake to 20 kcal/h for at least 2 days then returning to the standard intake over 2–3 days. There was no difference in days alive at 60 days after ICU discharge (primary outcome; difference, 4.9 days; 95%CI − 2.3 to 13.6; *P* = 0.19). However, day-60 survival was significantly higher with energy restriction than with standard intakes (91% vs. 78%, *P* = 0.002). A retrospective study involving 337 patients on MV supported this finding by showing that 50% energy restriction was associated with higher 6-month survival [61]. The data thus suggest that close monitoring of serum phosphate levels and energy restriction in the event of hypophosphatemia may improve outcomes in critically ill patients. RCTs are needed to assess this possibility.

**R-7.1:** A low protein dose of 0.2–0.9 g/kg/d (adjusted for BMI) should probably be given during the first ICU week in mechanically ventilated patients, instead of the standard protein dose of 1–1.3 g/kg/d (adjusted for BMI).

***Grade 2+, strong agreement***

**R-7.2:** The experts recommend returning to the standard protein dose (1.0–1.3 g/kg/d, adjusted for BMI) at the end of the first ICU week.

***Expert opinion, strong agreement***

A large RCT in 1329 patients on MV compared a high protein dose (≥ 2.2 g/kg/d) to the standard protein dose (≤ 1.2 g/kg/d) for up to 28 days [62]. The primary outcome was time-to-discharge-alive from the hospital up to 60 days after ICU admission. The cumulative incidence of survival to hospital discharge did not differ between the high-dose group and the usual-dose group. A post-hoc analysis of the subgroup with acute kidney injury (AKI) found that high-dose protein was associated with a longer time-to-discharge alive from the hospital and higher day-60 mortality [63]. These findings are supported by a recent metanalysis of 23 RCTs (3303 patients) comparing higher vs. lower protein doses in critically ill patients in mixed medical-surgical ICUs [64]. The mean protein doses were 1.49 ± 0.48 g/kg/d in the high-dose group and 0.92 ± 0.30 g/kg/d in the low-dose group. No significant differences were found for overall mortality, ICU LOS, hospital LOS, or the infection rate. In the subgroup with AKI, high-dose protein was associated with higher mortality.

A large RCT in 3044 patients compared 6 kcal/kg/d with 0.2–0.4 g/kg/d protein to 25 kcal/kg/d with 1.0–1.3 g/kg/d protein during the first ICU week [49]. The two primary outcomes were time to readiness for ICU discharge and day-90 all-cause mortality. By day-90, 628 (41.3%) of 1521 patients in the low group and 648 (42.8%) of 1515 patients in the standard group had died (absolute difference, − 1.5%; 95%CI − 5.0 to 2.0; *P* = 0.41). Median time to readiness for ICU discharge was 8.0 days [5.0–14.0] in the low group and 9.0 days [5.0–17.0] in the standard group (HR, 1.12; 95%CI 1.02–1.22; *P* = 0.015) [49]. Thus, higher protein doses do not improve patient outcomes and may increase mortality in those patients with AKI, while low protein doses decrease the time to readiness for ICU discharge.

**R8:** Supplemental parenteral nutrition (supplemental PN) should probably not be given before day 7 after ICU admission in critically ill adults who are unable to meet their nutritional needs with oral or enteral nutrition.

***Grade 2-, strong agreement***

The goal of supplemental PN is to increase protein and energy delivery when early EN alone does not allow achievement of the targets. Prospective observational studies have found no clinical benefits of early supplemental PN (< 48 h) compared to late supplemental PN (≥ 48 h) or early EN alone (started < 48 h) [65, 66]. In a placebo-controlled RCT, day-90 mortality was not lower with early EN plus supplemental PN (25 kcal/kg/d) than with early EN plus a placebo (10–20 kcal/kg/d) [67].

In the EPaNIC RCT, patients given late supplemental PN initiated on day 8 had shorter ICU stays compared to patients given early supplemental PN (initiated within 48 h after ICU admission) (HR, 1.06; 95%CI 1.00–1.13; *P* = 0.04) [48]. The median hospital stay was 2 days shorter in the late supplemental PN group. Early supplemental PN was associated with longer MV durations and higher frequencies of nosocomial infections, compared to late supplemental PN. Neither mortality nor functional status at hospital discharge differed between the two groups.

The Early-PN RCT included 1372 patients (82% on MV) with contraindications to early EN. Day-60 mortality (primary outcome) did not differ significantly between the early PN and standard-care groups [68]. Early PN was associated with a shorter MV time but not with ICU LOS or hospital LOS, compared to standard care.

The SPN trial included 305 patients who had received less than 60% of their energy target by day 3 and were randomized to either EN plus supplemental PN (100% of the energy target) or EN only, between days 4 and 8 [43]. Compared to EN only, supplemental PN was associated with a lower incidence of nosocomial infections (primary outcome) (HR, 0.65; 95%CI 0.43–0.97). No between-group differences were found for the other outcomes including mortality, MV duration, ICU LOS, and hospital LOS. However, this trial has limitations that affect the interpretation of its results. The physicians were not blinded, and the primary outcome, nosocomial infections, was not adjudicated. The incidence of nosocomial infections (27% in the EN group and 38% in the supplemental PN group) was higher than expected, given the relatively low mortality rates (ICU mortality, 7% and 5% in the EN and supplemental PN groups, respectively). Meta-analyses have not demonstrated any beneficial or detrimental effects of supplemental PN. However, the included studies were very heterogeneous regarding the periods they were conducted, patient populations, illness severity, and outcomes [69–71].

**R9:** Energy- and/or protein-enriched solutions should probably not be used in critically ill adults.

***Grade 2-, strong agreement***

Iso-caloric iso-osmotic EN solutions (1 kcal/mL) are the most widely used to meet energy goals. Hypercaloric solutions (> 1 kcal/mL) allow a higher energy intake without increasing the fluid volume in patients with gastrointestinal dysfunction or digestive intolerance, when fluid restriction is required, or for transitioning to oral feeding using intermittent EN (e.g., EN at night). In the TARGET RCT, patients were randomized to EN with a hypercaloric solution containing 1.5 kcal/mL or a standard isocaloric iso-osmotic solution [72]. The EN delivery rate was 1 mL/kg/h in both groups. The primary outcome was day-90 mortality. Compared to the standard solution, the hypercaloric solution resulted in a higher energy intake. The two groups were not different for day-90 mortality (26.8% in the hypercaloric group vs. 25.7% in the standard group; RR, 1.05; 95%CI 0.94–1.16; *P* = 0.41), MV duration, or infection rate. Regurgitation and vomiting were more common with the hypercaloric solution (19% vs. 16%), as was insulin use (56% vs. 49%). Of note, we are not aware of any study investigating the use of hypercaloric EN solutions with the specific goal of decreasing water and sodium intakes.

In a randomized feasibility trial, a high-protein EN solution (100 g/L) was associated with a higher protein intake compared to a 60 g/L solution, with no differences in energy intake, day-90 mortality, or EN duration [73]. No other RCT has compared patient outcomes with high-protein vs. standard EN solutions. The EFFORT-Protein trial compared high-dose protein (≥ 2.2 g/kg/d) to standard-dose protein (≤ 1.2 g/kg/d) started within 96 h after ICU admission and continued for up to 28 days. However, patients could receive any combination of EN or PN, intravenous amino acids, or enteral protein supplements to achieve protein-intake goals. Increasing the protein intake did not lower day-60 mortality [62]. Subgroup analyses suggested a harmful effect of high protein intake in patients with renal insufficiency and/or multiple organ failure [63]. The results of the TARGET Protein (NCT05647135) study should be available shortly.

***Micronutrients***

**R10.1:** The experts suggest the enteral or parenteral administration of micronutrient supplements in critically ill adults with insufficient micronutrient intakes (e.g., due to fasting, parenteral nutrition, or prolonged enteral nutrition < 1500 kcal/d) or with increased losses due to renal replacement therapy (RRT).

***Expert opinion, strong agreement***

**R-10.2:** The experts suggest that routine micronutrient assays are unnecessary in critically ill adults.

***Expert opinion, strong agreement***

Micronutrients, defined as essential nutrients required in trace amounts for health and survival, include vitamins and trace minerals [74]. Currently available PN solutions do not include micronutrients, which must therefore be provided as supplements. The amounts of micronutrients in EN solutions are those considered optimal for healthy individuals receiving 1500 kcal/d [75]. Factors associated with inadequate micronutrient intakes during EN include fasting for more than 4 h/d and receiving less than 50% of the target intake of 20–25 kcal/kg/d. Conversely, achieving at least 80% of this target may be associated with an improved micronutrient status [76, 77]. Micronutrient deficiencies require time to develop. RRT in critically ill patients may result in greater micronutrient loss. Although no clinical effects have been reported, supplementation decreases the frequency of abnormally low micronutrient levels [78–85]. The few studies of micronutrient dosages for critically ill patients do not support routine supplementation [86–91]. However, in patients with chronic alcohol abuse, B-vitamin and folate supplementation is recommended [92].

Several observational studies demonstrated a significant decrease in plasma micronutrient concentrations over the first ICU days. During the inflammatory response, plasma levels do not reliably reflect intracellular concentrations of trace elements and therefore cannot be used to diagnose deficiencies [93, 94]. Finally, no studies have demonstrated benefits from routine assays and subsequent administration of deficient micronutrients in critically ill patients.

***Management of enteral nutrition***

**R11: **The experts suggest reserving postpyloric feeding for critically ill adults with enteral nutrition intolerance refractory to appropriate management.

***Expert opinion, strong agreement***

Postpyloric or small-bowel feeding has the theoretical advantage of bypassing the potentially hypokinetic stomach of critically ill patients. A Cochrane meta-analysis of 14 trials (n = 1109 patients) showed that, compared to gastric feeding, postpyloric feeding was associated with a lower risk of pneumonia and increased nutrient delivery despite a longer time to EN initiation [95]. The two groups did not differ regarding mortality, MV duration, or ICU LOS. Similarly, a subsequent meta-analysis found lower pneumonia rates and higher energy and protein intakes but no difference in mortality or ICU LOS [96]. No large RCT is available. Moreover, compared to gastric feeding, postpyloric feeding is less physiological, and the need for specific expertise and equipment may delay EN initiation. The recent large RCTs on EN chiefly used gastric feeding [19, 47, 49, 62, 72]. Therefore, postpyloric feeding should be reserved for patients with persistent intolerance to gastric feeding despite appropriate management.

**R12:** The experts suggest that gastrostomy should not be used routinely instead of orogastric or nasogastric tubes for prolonged EN in critically ill adults.

***Expert opinion, strong agreement***

In critically ill patients, nasogastric or orogastric tube placement, typically performed by nursing staff, is the most common initial method of EN delivery. When prolonged EN is anticipated, percutaneous endoscopic gastrostomy tube insertion is a potential alternative to nasogastric/orogastric tubes. Six RCTs compared nasogastric and percutaneous endoscopic gastrostomy tubes for extended EN [97–102]. Five trials included fewer than 60 patients, the only exception being the FOOD trial (n = 321 [99]); only two ([99, 102]) had a multicenter design, and the overall methodological quality was low to very low. Two trials did not predefine a primary outcome [97, 100]. Five trials focused on stroke patients and only one investigated a heterogeneous population of critically ill patients [100]. Gastrostomy was performed within 48 h after ICU admission in two trials [101, 102] and later in four trials. Mortality data were available for only two trials, and the largest trial demonstrated no statistically significant difference in 6-month mortality [99]. Percutaneous endoscopic gastrostomy tubes may decrease the risks of regurgitation and VAP [98, 101]. These findings are consistent with a 2015 Cochrane meta-analysis that included most of the above-mentioned trials and other trials that were not confined to critically ill patients, including a trial in patients with dysphagia [103]. In a meta-analysis, however, neither mortality nor pneumonia rates differed between percutaneous endoscopic gastrostomy and nasogastric/orogastric tubes. Consequently, the decision to perform percutaneous endoscopic gastrostomy should be based on a careful assessment of individual patient benefits and risks, in conjunction with preferences of the patient or surrogates.

**R13:** Continuous enteral nutrition should probably be preferred over bolus enteral nutrition in critically ill adults.

***Grade 2+, strong agreement***

EN can be delivered continuously or intermittently [104]. Intermittent EN may consist in administration over 30–60 min every 4–6 h or in bolus delivery over 4–10 min four to six times a day. Continuous EN at a constant flow rate is widely used in general ICUs and should theoretically improve digestive tolerance and nutrient absorption compared to intermittent EN. Conversely, intermittent EN may promote increased patient mobility, stimulate protein synthesis, and augment gastrointestinal hormone secretion, potentially improving gallbladder contractions [104, 105]. Moreover, the fasting periods may benefit diurnal homeostasis and stimulate autophagy [106, 107]. However, aligning nutritional intakes on circadian rhythms has not been proven to produce clinical benefits [108]. In a meta-analysis of 14 RCTs, continuous and intermittent EN were not significantly different regarding gastrointestinal symptoms, intolerance to EN, nosocomial pneumonia, and glycemic control [109]. Additional high-quality RCTs are warranted.

**R14:** In critically ill adults, enteral nutrition should probably be continued until extubation, with no pre-extubation fasting period.

***Grade 2+, strong agreement***

Pre-extubation fasting to decrease the risk of aspiration during extubation is a common practice whose effectiveness has been challenged. In an observational, prospective, single-center study in 100 patients, gastric emptying as assessed by ultrasound was not different after fasting for more vs. less than 6 h [110]. An RCT in 24 patients undergoing bedside tracheostomy found no difference in aspiration or postoperative pneumonia rates between preoperative fasting durations of 6 h vs. 45 min [111]. In a pilot study in patients undergoing tracheostomy, compared to fasting, perioperative EN (n = 10) resulted in higher energy delivery with no increase in morbidity [112].

A larger, multicenter (22 ICUs), open-label, cluster-randomized, parallel-group, non-inferiority trial compared continued EN vs. a 6-h fasting period before extubation in 1130 critically ill patients [113]. The proportion of patients with the primary outcome of extubation failure (defined as a composite of reintubation or death by day 7) was 17.2% with continued EN and 17.5% with 6-h fasting (absolute difference, − 0.4%; 95%CI − 5.2 to 4.5%). VAP rates were similar between groups (1.6% vs. 2.5%; RR, 0.77; 95%CI 0.22–2.69). Importantly, the median time from the first successful spontaneous breathing trial to extubation was significantly shorter in the continued EN group vs. the 6-h fasting group. Consequently, the median time from the first successful spontaneous breathing trial to ICU discharge was also shorter in the continued EN group. ICU mortality was higher in the 6-h fasting group than in the continued EN group.

***Management of intolerance to enteral nutrition***

**R15:** The experts suggest either gradual or immediate achievement of the energy target, with monitoring of phosphatemia and digestive tolerance in critically ill adults receiving enteral nutrition.

***Expert opinion, strong agreement***

Compared to immediately supplying the energy target, increasing the EN supply gradually might decrease EN intolerance, which is common in patients on MV. A single RCT in 100 patients compared the two strategies in critically ill patients started on EN within 24 h of intubation [114]. Energy intakes and EN tolerance were monitored through day 7. The energy intake was significantly higher with immediate target achievement. The groups were not significantly different for vomiting rates, prokinetic agent use, or colonic distension. The Refeeding Syndrome RCT compared restricted vs. standard energy intakes for managing refeeding syndrome at the acute phase of critical illness in 339 patients [60]. In the restricted group, the EN supply was limited to 20 kcal/h for at least 2 days and subsequently adjusted based on serum phosphate levels. Over the 60-day follow-up period, the mean number of days alive after ICU discharge (primary outcome) was not significantly different between the standard and restricted groups (difference, 4.9 days; 95%CI − 2.3 to 13.6; *P* = 0.19). However, the number of patients alive on day-60 was higher in the standard group. Nosocomial infections were more common in the standard group. Thus, neither trial supports gradually achieving the energy target in all patients at the acute phase of critical illness. Of note, the Refeeding Syndrome RCT highlights the importance of energy restriction during the acute phase and of monitoring serum phosphate levels.

**R16:** Gastric residual volume (GRV) should probably not be monitored routinely with the goal of assessing enteral nutrition intolerance or preventing nosocomial pneumonia in critically ill adults.

***Grade 2-, strong agreement***

Critically ill patients often exhibit delayed gastric emptying, with an increase in gastric residual volume (GRV). A high GRV can lead to gastroesophageal reflux, regurgitation, and vomiting, which occur in up to 40% of patients on MV [115–117]. Although traditionally used to assess EN tolerance, GRV measurement lacks reliability, due to factors such as tube size and position, patient positioning, and methodological variability [118]. A high GRV is associated with an increased risk of VAP, but this relationship is unlikely to be causal.

In several studies, omitting GRV monitoring did not increase the VAP incidence despite higher vomiting rates [118–120]. A large multicenter RCT demonstrated no significant increase in VAP incidence (OR, 0.83; 95%CI 0.37–1.89) despite increased vomiting when GRV was not monitored [121]. Importantly, the absence of GRV monitoring promoted the achievement of energy targets by reducing unnecessary EN interruptions. No significant differences were observed for ICU LOS, mortality, or MV duration. Thus, routine GRV monitoring may be unnecessary in critically ill patients. Eliminating this practice from standard care may be warranted to optimize EN delivery without increasing adverse outcomes.

**R17.1:** For critically ill adults with persistent enteral nutrition intolerance despite adequate symptomatic treatment, the experts suggest decreasing the enteral nutrition delivery rate for a predefined period of a few hours rather than stopping enteral nutrition.

***Expert opinion, strong agreement***

**R17.2:** The experts suggest that causes of EN intolerance should be investigated before administering symptomatic treatment.

***Expert opinion, strong agreement***

EN intolerance is variably defined, usually based on a GRV > 250 mL or > 500 mL, with or without vomiting, regurgitation, abdominal distension, or diarrhea [115, 121–124]. EN intolerance is associated with higher mortality, pneumonia, and longer ICU stays, although causality is unproven [125–127]. Differentiating between proximal intolerance (stomach/small bowel) and distal intolerance (colon) is important. Given the variability in definitions, scoring systems have been developed to standardize the assessment. The European Society of Intensive Care Medicine (ESICM) developed the 2013 Acute Gastrointestinal Injury (AGI) scale, which scores gastrointestinal dysfunction from 0 (no symptoms) to 4 (failure with extra-digestive organ involvement) [128]. The Gastrointestinal Dysfunction Score (GIDS) was developed subsequently as an evolution of the AGI scale [129].

A systematic search for the cause of EN intolerance is crucial. A major concern is non-occlusive mesenteric ischemia (NOMI), for which EN can be a risk factor in critically ill patients receiving high-dose catecholamines and/or dobutamine [130]. Gastrointestinal ultrasonography may be a valuable adjunct to the physical examination as a means of estimating the GRV, identifying gastroparesis, and detecting early signs of NOMI such as portal venous gas [131, 132]. EN intolerance often leads to a reduction or interruption in EN delivery. No clear evidence exists to determine which of these two strategies is preferable [133]. Several management algorithms exist, but a consensus on the optimal treatment remains lacking. Stopping EN for the management of intolerance is a major cause of below-target feeding [124].

**R18.1:** Prokinetic agents should probably be used to decrease vomiting in critically ill adults with enteral nutrition intolerance.

***Grade 2+, strong agreement***

**R-18.2:** The experts suggest either erythromycin or metoclopramide, or both, as prokinetic agents.

***Expert opinion, strong agreement***

Prokinetic agents are widely used to improve tolerance to gastric EN in critically ill patients. Moderate-quality evidence supports their efficacy compared to a placebo or no intervention. In a meta-analysis, EN intolerance was significantly less common with prokinetics [134]. A subsequent meta-analysis supported beneficial effects in 10 of the 13 included studies [135]. Nonetheless, whether prokinetics improve clinically important outcomes such as mortality, ICU LOS, and the rate of pneumonia remains unclear [134, 135]. Metoclopramide and erythromycin are the most widely used prokinetic agents. Erythromycin may deserve preference [136–140]. In a meta-analysis of six studies, EN intolerance was significantly less common with erythromycin but not with other prokinetics [136]. However, metoclopramide is often preferred as the first-line agent due to concerns about ventricular arrhythmias and microbial resistance associated with erythromycin. Tachyphylaxis can occur with both drugs, limiting their long-term effectiveness. The ghrelin agonist ulimorelin has demonstrated similar efficacy to metoclopramide in promoting successful EN, without significant safety differences, but is not yet commercially available [141]. Combination therapies have been investigated, such as metoclopramide plus erythromycin or neostigmine, and were more effective than monotherapy in decreasing the GRV [142, 143]. The extent to which nutritional targets should be aggressively pursued in patients with EN intolerance, particularly during the first ICU days, remains debated. Further research is needed to define the optimal use of prokinetic agents in critically ill patients.

**R19.1:** In patients on mechanical ventilation who are receiving enteral nutrition and require prone positioning, the experts suggest continuing enteral nutrition during the prone periods.

***Expert opinion, strong agreement***

**R19.2:** The experts suggest that the nutritional protocol for patients managed with prone positioning should include prophylactic prokinetic agents and elevated head-of-bed position, to improve enteral nutrition tolerance.

***Expert opinion, strong agreement***

Prone positioning is a key treatment of acute respiratory distress syndrome (ARDS) [144]. Data on EN tolerance in critically ill patients turned in the prone position are conflicting. In some studies, EN intolerance and vomiting, leading to EN discontinuation and decreased intakes, were more common in the prone than in the supine position [145–147]. Other studies showed no clinically significant increases in EN intolerance or gastrointestinal complications in the prone position [148–150]. No studies have demonstrated associations of EN in the prone position with adverse effects on clinical outcomes including LOS, VAP, or mortality.

The implementation of nutritional protocols including the use of prokinetic agents and elevated head-of-bed position may improve EN tolerance. In a before-after study, after protocol implementation, the median EN volume delivered per day increased significantly [41]. Applying standardized EN management protocols may allow the delivery of greater EN volumes while also decreasing EN interruptions, regurgitation, and vomiting in patients treated with prone positioning [147, 151].

**R20.1:** The experts suggest the use of fiber-containing enteral nutrition products in critically ill adult patients with diarrhea.

***Expert opinion, strong agreement***

**R20.2:** There is insufficient evidence to recommend the routine use of fiber-containing enteral nutrition products to prevent diarrhea or other digestive complications in critically ill adults.

***Expert opinion, strong agreement***

Two types of fiber-containing EN products are currently available: one type contains both soluble and insoluble fiber and the other only soluble fiber (partially hydrolyzed guar gum). Fiber regulates intestinal transit, and depending on its type, influences viscosity, the fermentation rate, and/or fecal mass [152, 153].

In two RCTs, compared to a fiber-free EN, EN with partially hydrolyzed guar gum decreased the diarrhea severity score and, in patients with pre-existing diarrhea, decreased the number of diarrhea episodes [154, 155]. A meta-analysis of 19 studies showed lower diarrhea scores and a lower risk of gastrointestinal complications with fiber vs. no fiber [156]. The incidence of diarrhea (measured as the number of affected patients and/or the percentage of days with diarrhea) was significantly lower with vs. without fiber. Sub-group analyses showed similar results with soluble vs. mixed fibers. Fiber may protect from gastrointestinal complications, although fiber fermentation may contribute to abdominal distension in some patients [156, 157].

Fiber may also facilitate the faster achievement of energy targets and benefit glycemic control [155, 157]. However, available studies had limited sample sizes and heterogeneous populations, and produced only low-level evidence. Well-designed studies are needed before fiber-containing EN products can be recommended routinely and before the appropriate dosages for specific patient populations can be determined.

***Specific conditions***

*Immunonutrition*

**R-21:** Immunonutrition or specific immunonutrients should not be used in critically ill adults.

***Grade 1-, strong agreement***

Critically ill patients, particularly those with sepsis, may develop immunoparalysis, which increases susceptibility to hospital-acquired infections. Immunonutrition provides nutrients that influence immune function, tissue repair, and inflammatory responses. Key immunonutrients studied in the ICU include glutamine, arginine, taurine, omega-3 polyunsaturated fatty acids, fat-soluble vitamins (A, D, E, and K), water-soluble vitamins (B and C), and selenium.

While immunonutrition has shown efficacy in protecting against hospital-acquired infections in the perioperative setting, effects in critically ill patients remain controversial [158–160]. Omega-3 polyunsaturated fatty acids may provide benefits in patients with ARDS [161]. In contrast, supplementation with glutamine [162, 163] or vitamin C [164, 165] has been associated with increased mortality in critically ill patients. Selenium supplementation does not appear to significantly affect clinical outcomes [162, 166]. Clinical trials of immunonutrition showed either no benefit or potential harm, including increased mortality, compared to standard nutrition [167–169]. Studies used diverse nutrients, with different cellular targets, and enrolled a variety of patient populations, precluding general conclusions. Well-designed RCTs are needed. Based on the current evidence, the routine use of immunonutrients in the ICU cannot be recommended. As indicated above, the present guidelines do not apply to burn patients.

*Acute pancreatitis*

**R22.1:** In critically ill adults with acute pancreatitis and persistent organ failure, enteral nutrition should probably be initiated within the first week following ICU admission.

***Grade 2+, strong agreement***

**R22.2:** In critically ill adults with acute pancreatitis and persistent organ failure, enteral nutrition should be preferred over parenteral nutrition.

***Grade 1+, strong agreement***

**R22.3:** In critically ill adults with acute pancreatitis and persistent organ failure, initial nasojejunal tube feeding should probably not be prioritized over nasogastric tube feeding.

***Grade 2-, strong agreement***

An RCT in 205 patients with acute pancreatitis (30% with an APACHE II score > 13) compared early nasogastric tube feeding initiated within 24 h of randomization, which occurred within 24 h after presentation to the emergency department (early group) and an oral diet started 72 h after presentation (on-demand group). The primary endpoint was a composite of major infection or death at 6 months. The two groups did not differ significantly for the primary endpoint (RR, 1.07; 95%CI 0.79–1.44; *P* = 0.76), the infection rate, or mortality [170]. Other studies also found that early EN was not more beneficial than delayed EN or oral feeding [171, 172]. The studies vary widely regarding early EN timing (< 24 h or < 48 h), late EN timing (> 72 h, > 7 days, or combined oral feeding and EN), and required disease severity for inclusion. The data suggest that EN or oral feeding should be initiated within the first week after admission for severe acute pancreatitis. The evidence does not support routine early EN within 48 h. Meta-analyses showed lower mortality and fewer local and systemic infections with EN than with PN [16, 173]. The postpyloric route was not better than the nasogastric route regarding mortality, infections, organ failure, or ICU LOS in two other meta-analyses [174, 175]. Consequently, the nasogastric route should be preferred initially, as nasogastric tubes are easier to insert compared to nasojejunal tubes. These three recommendations are consistent with previous guidelines for the initial management of acute pancreatitis [176–179].

*Patients receiving noninvasive ventilation (NIV) or high-flow nasal oxygen (HFNO)*

**R23.1:** The experts suggest oral feeding with monitoring of swallowing for critically ill adults receiving noninvasive ventilation or high-flow nasal oxygen.

***Expert opinion, strong agreement***

**R23.2:** There is no evidence to recommend additional enteral nutrition or parenteral nutrition for critically ill adult patients receiving noninvasive ventilation or high-flow nasal oxygen during the first ICU week.

***Expert opinion, strong agreement***

Data on the nutritional management of patients receiving NIV or HFNO are limited. Dysphagia, nausea, anosmia, and the NIV mask can hinder both oral nutrition and EN. A retrospective study found that, during the first two days of NIV, patients either were not fed (57.8%) or received inadequate nutrition [180]. Intubation was more common in patients receiving PN or EN. Oral nutrition was protective compared to no nutrition. Furthermore, compared to no nutrition, EN was associated with higher mortality, whereas PN and oral nutrition were not. Compared to no nutrition, VAP was more common with PN and EN but not with oral nutrition. Two other studies found that oral nutrition in patients on NIV often failed to meet nutritional targets. In a prospective cohort study, NIV failure was more common in patients receiving EN via a gastric tube, although EN was administered to only 28.5% of those patients with NIV [181]. In a retrospective cohort study, EN was associated with higher rates of airway complications and longer NIV duration, but EN was started within 48 h in only 43% of patients [182]. Importantly, all these studies lacked comparison groups with similar admission diagnoses. An observational study in patients receiving HFNO found that 25% received only oral nutrition and 50% received EN [183]. Oral nutrition is possible with HFNO but can be limited by impaired swallowing at flows above 40 L/min, increased laryngeal vestibule closure, and disruption of lip-tongue synergy [184]. Adapting food textures; excluding or thickening liquids; and performing objective, standardized swallowing assessments before each oral feeding are often necessary.

***After ICU discharge***

**R24:** The experts suggest that a comprehensive nutritional and physical assessment be conducted before ICU discharge to develop a personalized nutritional and physical rehabilitation program designed to promote functional recovery.

***Expert opinion, strong agreement***

Multimodal rehabilitation requires a multidisciplinary approach involving intensivists, nutritionists, dietitians, rehabilitation therapists (including physiotherapists, adapted physical activity instructors, occupational therapists, and psychomotor therapists), speech therapists, psychologists, nurses, and nursing assistants. Multimodal rehabilitation in patients preparing for ICU discharge may improve nutritional intake and status, enhance physical function, and improve clinical outcomes. The RECOVER RCT (n = 240) in adults demonstrated that post-ICU hospital-based rehabilitation including enhanced physical and nutritional therapy and patient education was feasible and associated with improved patient satisfaction about various aspects of recovery [185]. Nutritional intakes of 30–35 kcal/kg/d and 1.2–1.5 g protein/kg/day, combined with appropriate physical activity, may promote recovery two months after ICU discharge, as evidenced by improvements in weight, muscle strength, muscle mass, walking speed, dysphagia, autonomy, and Barthel Index scores [186, 187]. The success of multimodal rehabilitation depends on setting individualized goals in collaboration with the patients and tailoring the protocol to maximize patient satisfaction while minimizing healthcare needs [185, 188, 189]. The EFFORT trial (n = 2088) demonstrated that protocol-guided, individualized nutritional support helped achieve protein and energy goals (in 75% of patients), thereby reducing the risk of adverse clinical outcomes and morbidity in hospitalized medical patients at nutritional risk, as defined by a Nutritional Risk Score (NRS)-2002 ≥ 3 [190]. However, to date, no RCTs in critically ill patients have directly compared multimodal rehabilitation to standard care regarding clinical outcomes after ICU discharge.

### Guidelines for children

**Initiation of nutritional support**

**Pediatric R1:** The experts suggest initiating enteral nutrition in critically ill children within 24–48 h after admission, in the absence of contraindications. Parenteral nutrition should probably not be initiated within 48 h of pediatric ICU (PICU) admission.

***Expert opinion, strong agreement***

No RCT has compared nutrition started within vs. after the first 48 h in the PICU. Early EN promotes and maintains gastrointestinal mucosal integrity and function [9]. Observational studies in the past decade have established the feasibility and safety of EN in critically ill children with medical or surgical diagnoses. The few contraindications of EN are not specific to pediatric patients and consist of a non-functional gastrointestinal tract, proximal digestive fistula, and coma without airway protection. Early start of EN was suggested for patients with stable hemodynamics [191, 192]. Early EN in PICU patients is variably defined. In a large retrospective multicenter study, early EN was defined as enteral delivery of 25% of the energy target over the first 48 h and was associated with lower mortality [193]. Early EN and early PN were compared in two RCTs. In the PEPaNIC trial, withholding supplemental PN for 1 week in the PICU led to fewer new infections and a shorter PICU LOS compared to early PN (within 24 h), even after adjustment for EN. Patients given no EN had similar outcomes to those who received EN. Early PN results in higher daily energy intakes than recommended. In an observational retrospective study, early PN in patients given no EN for the first 4 days was associated with higher mortality [194].

**Pediatrics R2:** The experts suggest enteral nutrition as the first-line approach in critically ill children, in the absence of contraindications.

***Expert opinion, strong agreement***

No RCTs comparing EN to PN in pediatric patients are available. A 1998 retrospective study compared the tolerance and complications of EN and PN in 29 children on ECMO, including 14 on EN only and 13 on PN only [195]. Achievement of predefined energy targets was not different between the two groups, and EN was not associated with any complications. A case–control study of 180 patients aged 2 months to 5 years and admitted to the PICU for respiratory distress compared outcomes with early EN (n = 90) and early PN (n = 90) [196]. Early PN was associated with higher mortality (27.1% vs. 11.1%, *P* = 0.01) and with higher rates of sepsis and VAP [197]. The many advantages of EN include a trophic effect on the gastrointestinal mucosa, less bacterial translocation, fewer infections, and lower costs. An international multicenter cohort study in 500 critically ill patients with a mean age of 4.5 years showed that EN was preferred based on the lower risk of infections and lower costs compared to PN [198]. Less than 9% of patients received PN in this study. The PEPaNIC RCT found greater morbidity in critically ill children receiving early supplemental PN compared to late supplemental PN [199]. Children with stable hemodynamics under vasoactive drug therapy or extracorporeal life support are often given EN, with a cautious approach and close monitoring of gastrointestinal tolerance [200].

**Nutritional needs**

**Pediatric R3:** The authors suggest using the standard Schofield equations in critically ill children and not using PICU-specific predictive equations to guide energy prescriptions.

***Expert opinion, strong agreement***

The accuracy of various predictive equations for estimating energy expenditure in critically ill children has been extensively investigated. Among these, the Schofield predictive equation has been recommended in previous guidelines and is widely used in pediatric intensive care units (PICUs) [201, 202]. However, only a limited number of equations have been developed specifically for ventilated, critically ill pediatric patients. One such equation incorporates clinical parameters such as diagnostic category, body temperature, and the day of PICU admission [203, 204].

Nevertheless, studies—including a systematic review—have demonstrated that more complex equations often perform worse than simpler predictive models, such as the Schofield or World Health Organization equations [205, 206]. More recently, a metabolic equation specifically designed for critically ill children, which requires accurate measurement of carbon dioxide production, has been reported [207]. While this method may be less feasible in children weighing less than 15 kg, it has demonstrated superior accuracy compared to conventional predictive equations and represents a promising avenue for future research.

In conclusion, given the limited precision and practicality of more complex predictive models, the Schofield equation remains the most pragmatic tool for estimating energy requirements in critically ill children.

**Pediatric R4:** The experts suggest extrapolating the recommendation on the use of indirect calorimetry in critically ill adults to critically ill pediatric patients.

***Expert opinion, strong agreement***

No PICU study has compared determination of the energy target by IC vs. the standard estimation using the Schofield equation. IC is the reference method for REE measurement but is challenging to use in critically ill children and is unavailable in most PICUs [208]. Contrary to the Deltatrac II®, which is no longer manufactured, none of the available devices has been validated in PICU patients [209]. Most PICUs determine energy targets using predictive equations, chiefly the Schofield equation [201] or WHO equation [210]. The Schofield equation is among the least inaccurate in critically ill children on MV but is not devoid of substantial bias [205]. Thus, at present, estimation of nutritional needs using the Schofield equation is the most pragmatic method.

**Pediatric R5:** In critically ill children, the experts suggest achieving the energy target determined using the Schofield equation by the end of the first PICU week and avoiding prolonged low-energy feeding.

***Expert opinion, strong agreement***

No study specifically designed to assess low-energy vs. standard-energy nutrition in critically ill children has been published. An RCT [199] and five observational studies assessed the effect of different energy intakes on clinical outcomes [198, 208, 211–213]. The energy targets varied, but the Schofield equation was often used to determine the standard energy intake. No clear definition of low-energy nutrition in PICU patients is available. In the PEPaNIC trial, the target of 50 kcal/kg/d was achieved on day 2 in the group started on supplemental PN within 24 h and on day 7 in the group given supplemental PN starting on day 8 [199]. The late supplemental PN group had fewer patients on RRT, shorter MV durations, and shorter PICU stays. Thus, the early provision of 50 kcal/kg/d, which is close to the REE or target provided by the Schofield equation, was deleterious. Similarly, an observational study showed that an energy intake > 110% of the REE measured by IC was associated with poorer outcomes [212]. In agreement with the PEPaNIC trial, observational studies showed lower mortality rates in children who received > 60% of the energy target or > 60 kcal/kg/d within the first 7–10 days in the PICU [208, 211, 213]. In the PIN1 multicenter study, mortality was higher in children who received less than 33% of the energy target within the first 7 days [198]. This result may be ascribable in part to the limited body reserves in children. Most of these studies were performed during the first PICU week in children who were on MV and sedated. As the clinical conditions change, the energy targets should be adapted, notably to the level of physical activity, cumulative energy deficit, and rehabilitation protocol. Overall, the data suggest better outcomes in patients with energy intakes close to the REE (provided by the Schofield equation) by the end of the first PICU week.

**Pediatric R6:** The experts suggest considering all causes of hypophosphatemia, including refeeding syndrome in severely malnourished children. If refeeding syndrome is suspected, the experts suggest that the standard protocol for temporarily decreasing nutritional intakes in non-critically ill children be followed.

***Expert opinion, strong agreement***

European guidelines for pediatric patients (but not specifically PICU patients) recommend screening for refeeding syndrome and achieving nutritional targets gradually in high-risk patients [214]. Other causes of hypophosphatemia must be considered. An ancillary study of the PEPaNIC RCT showed that refeeding syndrome was more common with early PN than with late PN [215]. Early refeeding syndrome was significantly associated with longer PICU and hospital stays. In a retrospective observational study in infants with bronchiolitis, lower phosphatemia at admission was associated with a longer MV duration [216]. No data in critically ill children are available regarding possible associations between reducing nutrition in the event of hypophosphatemia and patient outcomes. Given the lack of data specific to PICU patients, applying the standard protocol for non-critically ill children seems the best strategy.

**Pediatrics R7:** The experts suggest progressively increasing the protein intake by the end of the first PICU week to achieve the standard protein target of 1.5 g/kg/d in critically ill children.

***Expert opinion, strong agreement***

In critically ill children, a standard protein intake of 1.5 g/kg/d is recommended based on several studies [217–219]. A 2017 systematic review showed that daily protein intakes ranged from 0.67–1.5 g/kg/d in observational studies and 2.8–4.7 g/kg/d in RCTs in critically ill children [219]. Most of the studies showing benefits from higher protein intakes used EN. A protein intake > 1.1 g/kg/d was associated with a positive nitrogen balance and lower mortality, and these associations were strongest with intakes > 1.5 g/kg/d, in agreement with a previous systematic review [217]. A post-hoc analysis of the PEPaNIC trial sought to determine which macronutrient caused the harm seen with early PN [220]. Early administration of amino acids was associated with more infections, longer MV duration, and longer PICU LOS. A pilot RCT showed that protein-enriched EN helped to achieve the protein target compared to standard EN, with similar adverse-event rates and outcomes in both groups [221]. Thus, gradually achieving a protein target of 1.5 g/kg/d seems desirable.

**Pediatric R8.1:** Supplemental parenteral nutrition (supplemental PN) should be initiated after day 7 rather than on day 1.

***Grade 1+, strong agreement***

**Pediatrics R8.2:** The experts suggest considering the initiation of gradual supplemental parenteral nutrition after 48 h if enteral nutrition is expected not to achieve nutritional goals on day 7.

***Expert opinion, strong agreement***

The optimal time for PN initiation is controversial. PN is considered when EN fails to meet energy needs. The PEPaNIC RCT in 1440 critically ill children showed that delaying PN for one week was better than starting PN on day 1 regarding LOS, MV duration, and the incidence of infections [199]. Early EN was given in both groups. Late PN was started on day 8 when EN provided less than 80% of the energy target. An open-label, single-center RCT compared early PN (day 1) and late PN (day 4 in malnourished and day 7 in well-nourished patients [222]. EN was started later and progressed gradually as the PN intake was decreased. The primary outcome was the need for MV. MV was needed less often and, when needed, was given for shorter durations, in the early PN group. However, the high malnutrition rate in the trial population may limit the general applicability of these findings. No RCTs on PN timing focused on children with EN intolerance.

**Pediatric R9:** Energy and/or protein-enriched solutions should probably be preferred for critically ill children in whom a need for fluid restriction compromises the achievement of nutritional goals, notably after congenital-heart-disease surgery.

***Grade 2+, strong agreement***

Achieving fluid balance is challenging in critically ill children [223]. A meta-analysis found an association between fluid overload and higher in-hospital mortality [223]. In a case–control study of children after congenital-heart-disease surgery, patients whose cumulative fluid balance was ≥ 5% by day 2 had longer median MV durations, longer PICU stays, and longer hospital stays [224]. Most of the data on enriched solutions for PICU patients focused on children admitted for congenital heart disease surgery. Two RCTs and one prospective cohort study compared standard and protein-enriched EN in small, general PICU populations [221, 225, 226]. Feeding tolerance was not different between groups. Two meta-analyses of studies in congenital heart disease surgery children compared outcomes with solutions enriched in energy and proteins vs. standard solutions [227, 228]. Feeding intolerance, mortality, and the incidence of infections were not different but the enriched solutions were associated with shorter MV durations and shorter PICU and hospital stays.

**Micronutrients**

**Pediatric R10.1:** The experts suggest using micronutrient-enriched enteral nutrition or parenteral nutrition preparations in critically ill children with prolonged low-energy nutrition, fasting, or renal replacement therapy.

***Expert opinion, strong agreement***

**Pediatric R10.2:** The experts suggest not routinely performing micronutrient assays in critically ill children.

***Expert opinion, strong agreement***

Several studies show micronutrient deficiencies in PICU patients, and some suggest a correlation with greater disease severity. However, research on vitamin supplementation in the PICU and its relationships with clinical outcomes is limited. Vitamin D deficiency is present in up to 40% of PICU patients and may be associated with greater illness severity [229–232]. Vitamin C deficiency was found in 18% of PICU patients [233] and was associated with greater disease severity in patients after congenital-heart-disease surgery [234] or sepsis [235]. Selenium deficiency was associated with poorer clinical outcomes [236–238]. Vitamin E deficiency has also been described [239]. Thiamine deficiency was present in up to 30% of malnourished PICU patients and was associated with higher mortality [240]. An ancillary study of PEPaNIC data found deficient plasma levels of copper, zinc, and magnesium at admission in 4.7% of enrolled patients. Whether the link between micronutrient deficiencies and disease severity is causal has not been established [241]. In an RCT, a single, high dose of vitamin D in children admitted to the PICU for sepsis decreased the risk of septic shock [242]. High-dose vitamin D supplementation before congenital-heart-disease surgery with cardiopulmonary bypass decreased the risk of postoperative vitamin D deficiency without inducing adverse effects [243].

**Management of enteral nutrition**

**Pediatric R11:** Gastric enteral nutrition should probably be preferred over postpyloric enteral nutrition in critically ill children.

***Grade 2+, strong agreement***

Postpyloric EN was compared to gastric EN in three RCTs [244–246] and one observational study [247]. In one of the RCTs (n = 62), postpyloric feeding significantly increased the daily energy intake, whereas mortality, LOS, and aspiration rates were similar between groups [245]. A RCT in 40 patients found no difference regarding VAP occurrence [246]. The remaining RCT focused on aspiration, which was not significantly different between the two groups; of note, abdominal radiographs were more often required in the postpyloric group, which also had a longer time to EN initiation due to the more demanding tube-insertion technique [244]. In the case–control study, the percentage of the energy target met by day 3 was significantly higher in the postpyloric group, whereas mortality, time to EN initiation, and LOS were not different [247]. However, in these studies, GRV monitoring was part of the local protocol only in the gastric EN group. Moreover, in one of the RCTs, continuous EN was used in the postpyloric group and bolus EN in the gastric group, possibly biasing the results [246]. Despite the increased energy supply with postpyloric feeding, the absence of differences for other outcomes and greater ease of insertion of gastric tubes support the use of the gastric route.

**Pediatric R12:** Regarding the indications of gastrostomy in critically ill children, the experts suggest extrapolating the recommendations for non-critically ill children.

***Expert opinion, strong agreement***

No PICU studies have compared the nasogastric or orogastric route to gastrostomy for prolonged EN. In pediatric patients, the indications of gastrostomy depend on the underlying diagnosis. The 2021 updated ESPGHAN guidelines suggest gastrostomy for EN to avoid malnutrition in patients with severe chronic disease and also when swallowing is impaired when non-oral feeding is expected to be required for longer than 3–6 weeks [248, 249].

**Pediatric R13:** Either bolus gastric enteral nutrition or continuous gastric enteral nutrition should probably be used in critically ill children.

***Grade 2+, strong agreement***

Evidence is scant about whether continuous or bolus gastric feeding is best. Two small RCTs (n = 45 children) found no difference between continuous and bolus EN regarding the frequency of diarrhea or vomiting [250, 251]. EN was started earlier with bolus feeding. In an RCT, continuous EN achieved the energy target faster than did bolus EN, with no significant differences in vomiting and diarrhea but a higher frequency of EN intolerance in the intermittent group [252]. A small RCT compared continuous to bolus gastric EN in 25 intubated children [253]. Delivery was better with bolus EN and safety was similar with the two methods. An RCT in 147 patients on MV in seven PICUs showed faster achievement of the nutritional target with bolus than with continuous EN [254]. Neither the percentage of patients who achieved the target nor the serial oxygen saturation index differed between the two groups. These studies do not report data on GRV, anthropometric parameters, or biochemical markers. Three recent systematic reviews conclude that strong evidence is lacking to recommend either bolus or continuous EN for intubated PICU patients [255–257]. The clinical condition of the patient may, however, guide the choice between the two methods.

**Pediatric R14.1:** The experts suggest that gastric enteral nutrition need not be routinely interrupted before extubation in critically ill children.

***Expert opinion, strong agreement***

**Pediatric R14.2:** The experts suggest continuing enteral nutrition until extubation in critically ill children receiving postpyloric feeding.

***Expert opinion, strong agreement***

In two surveys conducted in the UK and France, respectively, most PICU healthcare professionals reported that a fasting period before extubation was standard practice [258, 259]. The underlying rationale is that gastric vacuity might make potential re-intubation safer and prevent aspiration and VAP. Fasting recommendations for elective surgery are often applied in the PICU, despite the absence of validation in this setting [260, 261]. Gut motility is impaired in critically ill children due to numerous factors, and gastric emptying may differ from that in patients preparing for elective surgery [262, 263]. Moreover, gastric clearance may differ with the continuous EN often used in the PICU compared to oral meals eaten by non-critically ill children. An RCT compared continuing or interrupting EN during the peri-extubation period [264]. No significant differences were found regarding vomiting, aspiration, VAP, or the reintubation rate. However, both groups were fed via the postpyloric route and the trial may have been underpowered. Data from adults suggest that continuing EN until extubation is safe, and the experts suggest this method also for critically ill children. Caution is recommended, however, in high-risk children (e.g., with a history of severe gastroesophageal reflux, a high risk of extubation failure, or difficult intubation).

**Management of intolerance to enteral nutrition**

**Pediatric R15:** The experts suggest increasing the enteral nutrition intake gradually in critically ill children and adjusting the progression rate according to tolerance.

***Expert opinion, strong agreement***

No RCTs have compared gradually increasing the EN intake to full-dose EN from the outset in PICU patients. A pilot RCT in 50 patients younger than 6 months admitted after congenital heart disease surgery with cardiopulmonary bypass compared rapid escalation vs. standard escalation to the EN target (27 vs. 63 h) regarding inflammation, insulin resistance, and morbidity [265]. No differences were found for cytokine or insulin levels, the insulin/glucose ratio, or the postoperative complication rate. PN should be increased gradually [266]. The parenteral glucose supply should be progressive given the disruption of the enteroinsular axis and decreased maximum glucose-oxidation capacity [267]. In a study of children who received no EN for 4 days, early PN was associated with significantly higher mortality [194]. In severely malnourished children, the nutritional intake should be increased slowly to avoid refeeding syndrome [268, 269].

**Pediatric R16:** The experts suggest that gastric residual volume (GRV) need not be monitored routinely in critically ill children.

***Expert opinion, strong agreement***

GRV monitoring was performed in most PICUs [270] as a marker for gastric emptying, which is impaired during critical illness [262]. A large GRV was thus taken to indicate EN intolerance. However, studies using gastric ultrasound or the acetaminophen absorption test showed that GRV did not correlate closely with gastric content in PICU patients [263, 271–274]. A single clinical observational study compared two PICUs with different local protocols: routine GRV monitoring was standard practice in one but not in the other [275]. The two groups were not significantly different for the percentage of the energy target achieved by day 4, the incidence of VAP, or the incidence of necrotizing enterocolitis. Data obtained in critically ill adults do not support routine GRV monitoring. The experts suggest that the same can be applied to critically ill children.

**Pediatric R17.1:** For critically ill children with enteral nutrition intolerance, the experts suggest considering a decrease in the enteral nutrition delivery rate and repeated assessments of tolerance to avoid prolonged underfeeding.

***Expert opinion, strong agreement***

**Pediatric R17.2:** The experts suggest that the causes of feeding intolerance should be investigated and treated.

***Expert opinion, strong agreement***

**Pediatric R17.3:** In critically ill children with enteral nutrition intolerance, the experts suggest a stepwise enteral nutrition advancement protocol.

***Expert opinion, strong agreement***

Most PICUs define EN intolerance as an increase in GRV, vomiting, diarrhea, and abdominal distension. No universally recognized definition exists, however. Two surveys showed that EN intolerance was among the main reasons for interrupting EN [259, 276]. No PICU studies have evaluated the effects of interrupting or decreasing EN in case of intolerance. Stepwise protocols have been shown to optimize EN escalation and to assist in the diagnosis and management of EN intolerance [277]. Interruptions for procedures and for EN intolerance are common barriers to achieving nutrient targets by EN [278]. Attention to these barriers in the PICU and efforts aimed at decreasing fasting times are desirable [279].

**Pediatric R18:** The available data do not allow the development of a recommendation regarding the use of prokinetics in critically ill children receiving enteral nutrition.

**Pediatric R19:** In critically children on mechanical ventilation receiving enteral nutrition and requiring prone positioning, the experts suggest continuing enteral nutrition during the prone periods.

***Expert opinion, strong agreement***

No studies have focused specifically on the relationships between prone positioning and nutrition in the PICU. However, an RCT comparing prone positioning vs. no prone positioning in PICU patients with acute lung injury showed that EN could be administered similarly in the two groups [280]. Similar findings were obtained in adults on MV [148]. Given the well-established effectiveness of prone positioning in improving oxygenation in patients with ARDS, the importance of maintaining adequate nutrition in critically ill children, and the good reported safety profile of EN in the prone position, the experts suggest that EN should be continued in children with ARDS who are placed in the prone position.

**Pediatric R20: **The experts suggest that enteral nutrition solutions containing fibers should be preferred over fiber-free enteral nutrition solutions in critically ill children.

***Expert opinion, strong agreement***

Of 97 identified studies, only two—an RCT and a 2023 observational study—specifically assessed the effects of fiber-enriched EN formula in critically ill children [281, 282]. The RCT found that an EN solution supplemented with 5.4 g of fiber/1000 mL (including inulin, fructo-oligosaccharides, and acacia gum), probiotics (*Lactobacillus paracasei* NCC2461 and *Bifidobacterium longum* NCC3001), and docosahexaenoic acid was well tolerated and safe and increased beneficial fecal bacterial groups [282]. The observational study showed that switching to a high-fiber formula (1 g fiber/100 mL) maintained stable concentrations of two key fecal short-chain fatty acids (propionate and butyrate) with no significant decrease in acetate during the PICU stay [281]. Stool frequency was reduced and stool consistency improved. In conclusion, the two available studies suggest potential benefits of fiber-enriched EN formulas in critically ill children. However, these studies are of moderate-to-low quality and are at high risk for publication bias. Dietary fiber consumption is already standard practice in neonates and infants receiving EN, as both breast milk and standard infant milk formulas contain dietary fibers. Moreover, there is no physiological rationale to exclude fiber from EN in critically ill children. Further research is needed to determine the optimal dosages and types of fiber in this population.

***Specific conditions***

*Immunonutrition*

**Pediatric R-21:** Immunonutrition or specific immunonutrients should probably not be used in critically ill children.

***Grade 2-, strong agreement***

Data on immunonutrition in the PICU are scarce and highly heterogeneous regarding both the types of immunonutrients used and the clinical endpoints. Although some micronutrients and immunonutrients blood levels are often low in critically ill children, the current evidence is insufficient to recommend routine supplementation with the goal of improving clinical outcomes. In an RCT, 50 patients were assigned to a standard EN preparation or to an EN preparation with added glutamine, ascorbic acid, selenium, zinc, arginine, and omega-3 fatty acids. Immunonutrition improved nutritional outcomes and the nitrogen balance but had no significant effect on inflammatory mediators, mortality, or PICU LOS [283]. An RCT in 98 patients compared standard PN to PN with added glutamine [284]. The latter significantly decreased inflammatory proteins but did not affect clinical outcomes. A larger RCT in 293 PICU patients assessed an EN preparation designed to prevent infections by supplying metoclopramide, high-dose selenium, zinc, and glutamine [285]. No significant differences vs. the control group were found for nosocomial infection rates, the incidence of sepsis or day-28 mortality. Of note, a substantial proportion of the study population was not severely ill. The ability of selenium supplementation to modulate inflammatory mediators has not translated into clinical benefits [236, 286]. A retrospective propensity-score-matched study in PICU patients with septic shock found lower mortality with vitamin C, hydrocortisone, and thiamine (9%) than with hydrocortisone alone or standard care [287]. These findings should be viewed with caution as the study was small and methodologically flawed.

*Acute pancreatitis*

The experts suggest extrapolating the recommendations for adults to children.

**R-22.1:** In critically ill children with acute pancreatitis and persistent organ failure, enteral nutrition should probably be initiated within the first week following ICU admission.

**R-22.2:** In critically ill children with acute pancreatitis and persistent organ failure, enteral nutrition should be preferred over parenteral nutrition.

**R-22.3:** In critically ill children with acute pancreatitis and persistent organ failure, nasojejunal tube feeding should probably not be given preference initially over nasogastric tube feeding.

***Expert opinion, strong agreement***

Acute pancreatitis is rare in childhood (13.2/100 000/y in the US) but is becoming more common [288, 289]. Most children with acute pancreatitis are admitted to general wards rather than PICUs. No studies on the timing of EN in children admitted to the PICU for acute pancreatitis are available. Guidelines issued in 2020 in the US for children admitted to general wards for acute pancreatitis include an expert opinion that early EN be given, in the absence of supporting evidence [289]. Early EN for acute pancreatitis is not widely used in French PICUs [288]. In a retrospective study, from the US, found that 48% of patients received EN, with a mean time to initiation of 2.3 days [290]. In a retrospective study of children admitted to wards for acute pancreatitis, early EN was associated with fewer PICU admissions, shorter LOS, and fewer progressions to severe acute pancreatitis [291]. Given the absence of studies on patients admitted to the PICU and of physiological considerations or clinical data of concern about this strategy, extrapolating recommendations for critically ill adults with acute pancreatitis to children seems reasonable.

*Patients receiving noninvasive ventilation or high-flow nasal oxygen therapy*

**Pediatric R23.1:** Oral or enteral feeding should probably be given to children with bronchiolitis admitted to the PICU due to a need for noninvasive ventilation (NIV) or high-flow nasal oxygen (HFNO).

***Grade 2+, strong agreement***

**Pediatric R23.2:** The experts suggest that oral or enteral feeding can be given to children admitted to the PICU for noninvasive ventilation or high-flow nasal oxygen.

***Expert opinion, strong agreement***

In Europe, intravenous hydration is variably used in patients with mild bronchiolitis. In a 2022 survey, 54% of physicians reported rarely or never using intravenous hydration [292]. A European multicenter retrospective cohort study reported that 77.8% of children receiving NIV were given nasogastric EN [293]. The frequency of gastrointestinal complications ranged from 4.78% to 20.0%, with emesis in 16.6% and pulmonary aspiration in 1.5% of the patients. Retrospective studies of children treated with HFNO provided conflicting data [294–298]. Importantly, these studies did not demonstrate an increased risk of aspiration or pneumonia in fed vs. fasted children. However, high-quality evidence on the specific impact of nutrition in children treated with NIV or HFNO in the PICU is currently lacking.

***After ICU discharge***

**Pediatric R24:** Experts suggest integrating nutritional assessments in the post-PICU follow-up program and considering catch-up growth while setting nutritional goals.

***Expert opinion, strong agreement***

Over the past three decades, advances in PICU management have substantially improved survival and other outcomes. The treatment goal is no longer only to ensure survival but also to provide a good health-related quality of life. In a single-center study, the body-mass-index (BMI) Z-score decreased by > 1SD and > 0.5 SD in 10.2% and 27.8% of PICU patients, respectively [299]. After PICU discharge, most patients recovered normal BMI Z-scores within 3 months [130]. Research has established a link between growth and improved long-term outcomes. A 2014 multicenter cohort study evaluated a home monitoring program applied between two surgical stages in patients with hypoplastic left heart syndrome [300]. The program included daily recordings of weight and intakes and was associated with improvements in both mortality and quality of life.

## Data Availability

Not applicable.

## Abbreviations

95%CI: 95% Confidence interval
AKI: Acute kidney injury
ARDS: Acute respiratory distress syndrome
BMI: Body mass index
EN: Enteral nutrition
GRV: Gastric residual volume
HFNO: High-flow nasal oxygen
HR: Hazard ratio
IC: Indirect calorimetry
ICU: Intensive care unit
LOS: Length of stay
MV: Mechanical ventilation
NIV: Noninvasive ventilation
NOMI: Nonocclusive mesenteric ischemia
OR: Odds ratio
PICU: Pediatric intensive care unit
PN: Parenteral nutrition
RCT: Randomized controlled trial
REE: Resting energy expenditure
RR: Relative risk
RRT: Renal replacement therapy
VAP: Ventilator-associated pneumonia
